# Model-based optimized steering and focusing of local magnetic particle concentrations for targeted drug delivery

**DOI:** 10.1080/10717544.2020.1853281

**Published:** 2020-12-21

**Authors:** Rikkert Van Durme, Guillaume Crevecoeur, Luc Dupré, Annelies Coene

**Affiliations:** a Department of Electromechanical, Systems and Metal Engineering, Ghent University, Gent, Belgium; b EEDT Decision & Control, Core Lab Flanders Make, Ghent, Belgium; c Cancer Research Institute Ghent, Ghent, Belgium

**Keywords:** Magnetic drug targeting, magnetic nanoparticles, modeling, optimal control, model order reduction, targeted delivery

## Abstract

Magnetic drug targeting (MDT) is an application in the field of targeted drug delivery in which magnetic (nano)particles act as drug carriers. The particles can be steered toward specific regions in the human body by adapting the currents of external (electro)magnets. Accurate models of particle movement and control algorithms for the electromagnet currents are two of the many requirements to ensure effective drug targeting. In this work, a control approach for the currents is presented, based on an underlying physical model that describes the dynamics of particles in a liquid in terms of their concentration in each point in space. Using this model, the control algorithm determines the currents generating the magnetic fields that maximize the particle concentration in spots of interest over a period of time. Such an approach is computationally only feasible thanks to our innovative combination of model order reduction with the method of direct multiple shooting. Simulation results of an in-vitro targeting setup demonstrated that a particle collection can be successfully guided toward the targeted spot with limited dispersion through a surrounding liquid. As now present and future particle behavior can be taken into account, and non-stationary surrounding liquids can be dealt with, a more precise and flexible targeting is achieved compared to existing MDT methods. This proves that the presented methodology can bring MDT closer to its clinical application. Moreover, the developed model is compatible with state-of-the-art imaging methods, paving the way for theranostic platforms that combine both therapy as well as diagnostics.

## 1. Introduction

Targeted drug delivery is playing an increasingly important role in the field of cancer and disease treatment. Its goal is to specifically guide and release therapeutics toward a diseased region in the human body. This leads to an improved therapy efficacy while mitigating potential adverse effects that occur when drugs are injected nonspecifically (Widder et al., 1980; Bae & Park, 2011). Targeted drug delivery covers a broad range of disciplines such as *in-vivo* biochemical and physical drug release mechanisms, implantable systems, nanocarrier applications, etc. In this field also the subject of magnetic drug targeting (MDT) is researched, in which carriers in the nano- or micrometer size range are magnetically responsive, allowing them to be non-invasively manipulated or triggered by external magnets for all kinds of therapeutic or diagnostic purposes (Lübbe et al., 2001; Pankhurst et al., 2003, 2009; Price et al., 2018; Liu et al., 2019; Mirza, 2020). By coating these magnetic nano- (MNP) or microparticles with chemotherapeutic agents, the treatment of tumorous tissue is enabled. The particles’ small size makes it possible to reach targets deep inside the body and get behind cellular and tissue barriers. Furthermore, the particles’ magnetic response is measurable by sensitive sensors so that they can be localized with dedicated imaging methods such as magnetorelaxometry or magnetic particle imaging (Gleich & Weizenecker, 2005; Wiekhorst et al., 2012; Coene et al., 2017). Preclinical MDT trials exploiting these advantageous properties have been executed on small animals and humans to a limited extent (Lübbe et al., 1996; Chertok et al., 2011; Al-Jamal et al., 2016), because a full clinical *in-vivo* application of MDT is still subject to numerous open challenges, as stated by Shapiro et al. (2015). One of those challenges is the deep tissue targeting of single particles or particles distributed in a fluid (ferrofluids), the latter being a more intricate task than the former. Deep tissue targeting in this context means bringing particles toward lesion sites through tissue and anatomical barriers, and successively retaining or focusing them locally to ensure effective therapy for a period of time. To achieve this, there is a need for (1) suitable (biochemical) preparation and selection of carriers to make them biologically compatible, (2) real-time imaging modalities to localize carriers, (3) precise models apprehending *in-vivo* carrier motion, and (4) dynamical control algorithms to activate external magnet systems (Shapiro et al., 2015). This work formulates, implements and simulates a model-based control algorithm to address the control-related challenge (4).

Models for MNP motion and retention in fluids and biological environments have been investigated extensively (Grief & Richardson, 2005; Rotariu & Strachan, 2005; Furlani & Ng, 2006; Cherry et al., 2010; Furlani, 2010; Nacev et al., 2010; 2011; Tehrani et al., 2015; Kolitsi & Yiantsios, 2020). By examining the forces and motion of a single particle in liquids and Y-shaped channels under magnetic, fluidic, interacting and other forces, insights are gained about the parameters that affect it (Rotariu & Strachan, 2005; Furlani & Ng, 2006; Cherry et al., 2010; Furlani, 2010; Tehrani et al., 2015). Single particle force models however do not take into account diffusion, blood convection, boundary layer formation, extravasation and other phenomena occurring when multiple particles are distributed throughout vasculature networks and tissue (membranes). These different mechanisms and stages of in-vivo particle transport have been addressed in an integrated fashion in Refs. (Grief & Richardson, 2005; Nacev et al., 2010, 2011; Kolitsi & Yiantsios, 2020). Rather than single particles, concentrations or collections of particles are located, via their spatial distribution. This procedure is more involved and requires solving partial differential equations (PDE) such as the advection-diffusion equation with appropriate boundary conditions for each environment. The hereby significantly increased accuracy of the result comes at the cost of extended simulation times. In our effort to incorporate a model within a control algorithm, we need to find a middle ground between model fidelity and computational execution time. Therefore an *in-vitro* two-dimensional (2-D) experiment with uniform fluid characteristics and without complex structures is simulated to serve as an *in-silico* proof-of-concept of our control algorithm. We convert the advection and diffusion dynamics of particles in a uniform fluid to state-space equations in terms of square grid elements (called *pixels*, or *voxels* in 3-D, in agreement with MNP imaging literature), each containing a certain particle concentration. Not only does this allow for a fast evaluation of the concentration distribution over a time interval, it also elaborates on state-of-the-art MNP imaging modalities that resolve the particle concentrations in pixels/voxels. To the authors’ knowledge this way of modeling the concentration dynamics has not been reported before and we believe that together with potential fast particle-imaging applications, it may further enhance modeling and control of particle motion *in-vitro* and *in-vivo*.

By Earnshaw’s theorem, it is impossible for static magnetic fields to maintain magnetic dipoles in a stable stationary equilibrium (Earnshaw, 1842), implying that small particles cannot be focused in one spot merely by a constant magnetic field. This problem can be tackled in different ways, for example by making the magnetic fields time-dependent. This is where dynamical control algorithms come into play: by monitoring the fields generated by (electro-)magnets based on the location of particles in one time interval, decisions can be made in order to manipulate the particles’ motion in a next time interval by applying new electromagnet currents. These decisions are based on the minimization of a so-called *cost function* containing metrics of interest such as particle movement time, energy consumption, particle spreading, etc. Reported control strategies have treated the guiding or steering of single particles or droplets (Probst et al., 2011; Komaee & Shapiro, 2012; Khalil et al., 2016) and distributed ferrofluids by means of electromagnets (Shapiro, 2009; Komaee, 2017; Antil et al., 2018; Liu et al., 2020). An in-depth review of many prior magnetic drug targeting solutions with a focus on control systems for magnetic fluids was provided by Nacev et al. in 2012 (Nacev et al., 2012). Regarding the control of distributed ferrofluids, methods have been proposed to concentrate particles at a certain point, either by choosing or rotating magnetic fields until particles are focused at the destination (Shapiro, 2009; Liu et al., 2020), or by moving the ferrofluid from the initial to the final point along a straight line (Nacev et al., 2012) or a chosen curve (Komaee, 2017) while minimizing the spreading of the particles and dissipated energy. Most of these methods only provide optimal results that are local in time, in the sense that they optimize for the current distributions at that time instant and do not account for future ferrofluid behavior, making their control algorithms less accurate and efficient. Komaee discussed a method to overcome this using *optimal control* (Komaee, 2017). His cost function is formulated such that the particle dispersion and the required movement time be minimized, subject to the constraint that the center of mass remains on a desired trajectory. Another optimization-based approach was elaborated by Antil et al., who aimed at moving a domain of particles from an initial to a desired location by keeping the forces on particles in this domain almost constant, thereby minimizing particle spreading (Antil et al., 2018). We use the optimal control framework as well to ensure optimality over a period of time, but do not require a predefined trajectory between the beginning and end point of the ferrofluid, which is not always available beforehand or optimal. Not only can the spread of the particles be minimized as before, but now for the first time can the concentration at the target location be maximized, thanks to our voxel-based modeling and control. The resulting control method has a higher flexibility than existing methods, since it allows the choice of targeted spots while coping with space-dependent flow velocities of the surrounding fluid, if present. This was accomplished with the direct multiple shooting (DMS) formalism, one of the methods used in optimal control that lends itself excellently to solving our problem (Diehl et al., 2006).

The computational time to execute the DMS algorithm increases strongly with increasing numbers of grid points. To maintain a certain level of accuracy in the concentration distribution and at the same time feasible computational costs, the notion of *model order reduction* was introduced (Schilders et al., 2008; Baumann, 2013). It transforms the problem into a smaller sized one without significantly compromising the accuracy of a solution that would otherwise require infeasibly large computation times. This reduced-order method for boosting calculation speeds may further pave the way toward real-time imaging/targeting platforms in which quick measurements help the targeting algorithm to achieve its goal. This is to our knowledge the first time DMS and model order reduction are introduced to the magnetic targeting problem.

The structure of this text is as follows. We first describe a commonly employed 2-D targeting setup used in our simulations. Then in Section 2.2, a dynamical model is developed for the control strategy that takes into account the magnetic and fluidic forces and the advection-diffusion equation. This model is then discretized to obtain a state-space formulation in terms of grid concentrations. Next, in Sections 2.3 and 2.4 the developed model is applied to set and solve the control problem associated with guiding a collection of particles toward a target location through the fluid medium. Finally, it is shown in the Results section that the proposed method provides efficient and accurate targeting performance while being more flexible with target voxels, cost functions and flow velocities than existing procedures.

## 2. Materials and methods

### 2.1. Targeting setup

**Figure 1.**
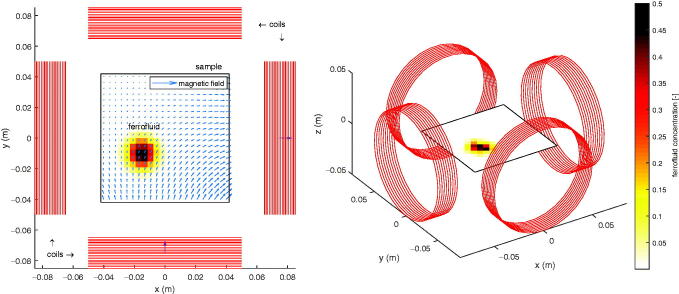
Setup of 4 coils (red circles) and 2-D sample (square). The colorbar shows the dimensionless measure for the concentration of nanoparticles in each square voxel. The superposition of the magnetic fields generated by the 2 coils (down and left) is indicated by blue arrows.

A detailed review of magnet systems for targeted drug delivery was given by Liu et al. (2019). Many MDT setups consist of multiple electromagnets (coils) surrounding the targeted region. Electromagnets enable to readily increase or decrease the magnetic field by altering the supply voltage. Since it is the current that directly affects the magnetic field and gradient and hence the forces acting on the particle, the time delay between voltage and current for a resistor-inductor network and mutual inductance should be taken into account. The voltage control is possible by means of digital-to-analog converters connected to DC amplifiers that convert a computer signal to the appropriate voltage level (Probst et al., 2011). Furthermore, as will be discussed, the force on magnetic particles is directed toward the point with the largest magnetic field magnitude. Turning on a single magnet thus makes the particles move toward the magnet since the field is larger close to the magnetic source. Therefore it is useful to put electromagnets on all sides of the sample. Setups for the steering or guiding of magnetic particles in two spatial dimensions (2-D) have been built and/or simulated with 4 [26, 28] or 8 [29, 31] electromagnets in the targeting plane. For particle movement in two spatial dimensions, at least 4 electromagnets should be used, and the more magnets, the more controllable the fields and gradients and thus the movement get. Our presented approach will be applied in simulation to a setup with realistic values as a proof-of-concept of the methodology. Four coils modeled as infinitely-thin circular wires with multiple turns are placed symmetrically with their axes in the same plane around a square sample of 8 cm × 8 cm with impermeable boundaries (petri dish) containing a liquid and biocompatible magnetite nanoparticles (Fe_3_O_4_ core, diameter of 400 nm, saturation magnetization $M_{s}=4.78\cdot 10^{5}$ A/m). Such a setup facilitates the validation of our model and control algorithm. The impermeable walls allow to keep the liquid and particles in one place. If flow rates are required, a tube and pump system can be added, as reported in Radon et al. (2017). The sample space is subdivided in identical square or cubic elements. These are called voxels throughout this work, as the sample can be seen as one layer of 3-D cubes with each cube containing a uniform concentration of particles. Since this layer lies in the same plane as the coils, the resulting force components directed out of this plane are negligible in this setup and particle motion will only be in 2-D (i.e. the *xy*-plane). Extension to 3-D is possible by adding more coils, but is not treated in our current discussion. Each voxel carries a certain amount of MNPs that is normalized and thus indicated by a dimensionless number (instead of e.g. in mg/ml), depicted schematically in Figure 1. As an illustration, two coils are carrying a current and generate a magnetic field by superposition.

In the next section, a model for the particle motion is developed, based on which the dynamic control algorithm operates.

### 2.2. Dynamical model

Based on a single magnetic particle’s equation of motion, the dynamics of particle ensembles can be described. This is done by combining the effect of diffusion and advection with the forces acting on the particles.

Consider the forces on a single particle in a fluid (Gerber et al., 1983; Cherry et al., 2010)

(1) $$m_{p}\frac{dv}{dt}=F_{m}+F_{D}+F_{L}+F_{g}+F_{b}+R+d$$

where *m_p_* is the particle’s mass, ***v*** the particle’s velocity, $F_{m}$ the magnetic force, $F_{D}$ the hydrodynamic drag force, $F_{L}$ the hydrodynamic lift force, $F_{g}$ the gravitational force, $F_{b}$ the buoyant force, ***R*** a random Brownian force, and ***d*** are particle interaction forces.

For the magnetic force on a particle, the magnetic charge model $F_{m}=\left(m\cdot \nabla \right)B$ is used (Boyer, 1988). Small magnetic particles, in ranges from nanometers to hundreds of nanometers depending on the type of material, have a magnetic behavior of a single domain and can as such be treated as magnetic dipoles. This means that m=VM, where ***M*** is the magnetization and *V* is the particle core volume. The uniaxial anisotropy of the particles generally causes their magnetization to be along one preferred direction in the absence of external magnetic fields, whereas it flips away toward the external field direction when this external field is sufficiently high (Kodama, 1999; Batlle & Labarta, 2002; Pankhurst et al., 2003). Depending on the effective uniaxial anisotropy $K_{\text{eff}},$ the behavior of the magnetization magnitude *M =* **||*M*||** is described by the commonly used Langevin function $L(x)=\coth(x)-\frac{1}{x},$

(2) $$M(H)=M_{s}L\left(\frac{\mu_{0}M_{s}VH}{k_{B}T}\right)$$

or other functions such as $M(H)=M_{s}\tanh\left(\frac{\mu_{0}M_{s}VH}{k_{B}T}\right),$ where *M_s_* is the saturation magnetization of the particle material, *k_B_* is the Boltzmann constant, *T* is the particle temperature, and *H* is the magnitude of the externally applied magnetic field ***H*** (Carrey et al., 2011). In this text, it is assumed that the single particle magnetic moment is aligned with the external field, thus m=g(H)H, where the function *g*(*x*) is to be determined based on the magnetic behavior and physical properties and/or the function in Equation (2). Plugging this into the magnetic charge model with ∇×H=0 (Ampère’s law) and $B=\mu_{0}H$ (Forbes et al., 2003; Grief & Richardson, 2005)

(3) $$F_{m}=\left(m\cdot \nabla \right)B=\mu_{0}\left(g(H)H\cdot \nabla \right)H=\mu_{0}g(H){\left[\frac{\partial H}{\partial x}\right]}^{T}H=\frac{\mu_{0}}{2}g(H)\nabla (H^{2})$$

From this it is inferred that the force on the particle is directed toward regions with a higher field magnitude. As mentioned before, the sources of the magnetic field and gradient are infinitely-thin current-carrying circular coils with multiple turns. The field is calculated by transforming the semi-analytical expressions from cylindrical coordinates to cartesian coordinates (Smythe, 1967; Simpson et al., 2001; Burke & Diamond, 2012). These expressions are in terms of the complete elliptic integrals of the first and second kind *K*(*m*) and *E*(*m*). In order to calculate the field gradient, we used their derivatives $\frac{dK(m)}{dm}=-\frac{K(m)}{2m}-\frac{E(m)}{2m(m-1)}$ and $\frac{dE(m)}{dm}=\frac{E(m)-K(m)}{2m}$ and a coordinate transformation.

The hydrodynamic drag force is drawn from Stokes’ law of viscous drag (Stokes, 1851; Pankhurst et al., 2003; Furlani & Ng, 2006)

$$F_{D}=-6\pi \eta R_{p}(v-v_{f})$$

where *η* is the dynamic viscosity of the surrounding fluid, *R_p_* is the hydrodynamic radius of the particle, $v_{f}$ is the velocity of the surrounding fluid.

Numerical simulations conducted by Cherry et al. (2010) point out that the effects of the random Brownian diffusive force ***R*** and particle interactions ***d*** are minor and can be neglected in the equation of motion. With particles in the sub-micron size range and low flow rates, the gravitational, buoyant, and lift force in Equation (1) are also negligible against the hydrodynamic drag and magnetic forces. The effect of particle inertia is omitted because of the particle’s low mass and the relatively large drag, making the acceleration period negligible (Roux, 1992). Hence the average velocity changes almost instantaneously with the magnetic force, thus $v=\zeta^{-1}F_{m}+v_{f}$ where $\zeta =6\pi \eta R_{p}$ is called the viscous drag and its inverse the mobility. It is noted that except for the particle interactions ***d***, these force components can still be added effortlessly to the model without a significant increase in computational cost.

As already mentioned, biomedical targeting applications usually consist of large numbers of particles distributed throughout fluids. The continuity equation describes the mass transport of the particles (Pedlosky, 2013)

$$\frac{\partial c}{\partial t}+\nabla \cdot j=S$$

where *c* is the particle concentration or the amount of particles per unit volume, ***j*** is the total flux of particles and *S* is a volumetric source for *c*. *S* = 0 when no particles are added to the system during the considered time interval. The flux consists of a diffusive component $j_{D}$ and an advective component $j_{A},j=j_{D}+j_{A}.$ The effect of Brownian diffusion is given by Fick’s law $j_{D}=-D\nabla c$ with *D* the diffusion coefficient (Fick, 1855). Advection of particles in the fluid is taken into account in $j_{A}=vc$ (Bejan, 2013). The result is the well-known advection-diffusion equation (Gerber et al., 1983; Takayasu et al., 1983; Grief & Richardson, 2005; Shapiro, 2009)

(4) $$\frac{\partial c}{\partial t}=\nabla \cdot \left(D\nabla c\right)-\nabla \cdot \left(vc\right)$$

This equation applied to magnetic particles in the human body was discussed in more detail by Nacev et al. (2011). It mentions other mechanisms contributing to or inhibiting MNP movement. Firstly the Stokes drag force does not include variations from wall effects, tissue and membranes. Moreover, particle chaining and agglomeration in blood vessels may influence the MNP behavior. Finally, in reality Brownian diffusion is not the only form of diffusion: blood cells scattered in the plasma interact with nanoparticles and increase the particles’ diffusion rate. This is referred to as shear-induced diffusion (Wang & Keller, 1985; Grief & Richardson, 2005). Inaccuracies in these parameters are important sources of error in *in-vivo* models. Some of these effects can still readily be added, others would unnecessarily complicate the model with regards to the proof-of-concept of our control methodology, discussed in the next section.

In order to find the spatial concentration distribution of particles in a time interval, it is required to solve the PDE in Equation (4) with known boundary values and initial values. For a petri dish with a ferrofluid, the no-flux boundary condition $n\cdot \left(j_{D}+j_{A}\right)=0,$ where ***n*** represents the normal outwards to the domain at the edge ∂Ω, means that no particle can cross the boundary and the total particle mass in the domain Ω is constant. This corresponds to n·(D∇c−vc)=0 (Komaee, 2017). This problem can be solved numerically using finite elements or finite differencing techniques. By approximating the sample space as a 2-D grid of voxels with length *Δx*, each with a certain concentration $c_{i,j}=c(x_{i},y_{j})=c(x_{0}+i\Delta x,y_{0}+j\Delta x),$ and applying $\frac{\partial c_{i,j}}{\partial x}=\frac{c_{i+1,j}-c_{i-1,j}}{2\Delta x}$ and $\frac{\partial^{2}c_{i,j}}{\partial x^{2}}=\frac{c_{i+1,j}-2c_{i,j}+c_{i-1,j}}{\Delta x^{2}}$ in (4), we eventually obtain

(5) $$\dot{c}=Ac$$

with ***c*** the vector of concentrations of each voxel and *A* a matrix encompassing magnetic and fluid dynamics. This equation can be solved numerically using explicit or implicit time integration methods. Depending on the spatial discretization scheme, the solution may contain oscillations when particle concentrations are large at domain boundaries for small diffusion coefficients compared to the advection effect. Methods to overcome these oscillations have been discussed in prior research and can be adopted in our approach (Nacev et al., 2011; Antil et al., 2018). Such high particle concentrations at the boundaries are circumvented by our control algorithm as it allows setting lower and upper bounds to concentrations in voxels of choice (as explained in Section 2.3 and 3). We use a 30 × 30 grid of voxels with equal length Δx=0.08/30 ≈ 2.7 mm for the spatial discretization and the fourth-order Runge-Kutta method for the time integration of (5).

The advantage of finite-difference or finite-volume methods in the field of magnetic targeting is that the domain is subdivided in voxels, which is the starting point of many MNP imaging modalities. As these imaging procedures use measurement data to reconstruct actual voxel concentrations, this information could be transferred immediately to a magnetic targeting routine to improve its performance. This direct link between targeting and imaging has to our knowledge not been approached in that manner. On top of that, our transformation of the problem to a state-space equation is particularly useful in the realm of dynamic optimization, which serves the steering and focusing of collections of particles at single locations. This is the topic of next section.

### 2.3. Dynamic optimization in a targeting problem

As discussed in the introduction, in magnetic drug targeting one of the main goals is to use the magnetic response of particles to manipulate their movement toward a desired location for continued localized therapy in the human body. The idea behind this strategy is that the magnetic force and thus the particle movement are *controllable*. Indeed, by merely adapting the current in electromagnets, the magnetic field and gradient and hence the magnetic force in a point in space is altered accordingly. In a way that has been made clear in the previous section, this affects the motion of the particles through their environment. If the current levels are changed *dynamically* based on modeled or measured locations of particles, a more accurate and satisfactory targeting performance can be achieved, greatly improving therapy efficacy. This brings us to the field of control theory and more specifically *optimal control*. In optimal control one aims to find a control for a dynamical system such that an objective or cost function is minimized over a period of time (Leunberger, 1979). This dynamic optimization can be executed with a variety of numerical techniques. Applied to our targeting problem, the dynamical system is described by Equation (5), the control is the electromagnet currents (also called *inputs*), and an example of an objective function is the concentration of particles in target voxel(s). By maximizing this concentration over a period of time, it is made sure that enough particles are present at every time instant in these voxel(s), enhancing therapeutic processes. The optimal control problem is formulated as (Diehl et al., 2006):

*find*

(6) $$\left\{u^{*},t_{f}^{*}\}=\arg\min_{{}_{u,t_{f}}}J=\arg\min_{{}_{u,t_{f}}}\int_{0}^{t_{f}}l(x(t),u(t))\;dt+e(x(t_{f})\right)$$

*subject to*

$$\begin{array}{ccc} x(0)-x_{0}=0 & & \text{fixed}\;\text{initial}\;\text{value}\\ \dot{x}(t)-f(x(t),u(t))=0 & t\in [0,t_{f}] & \text{ODE}\;\text{model}\\ h(x(t),u(t))\geq 0 & t\in [0,t_{f}] & \text{path}\;\text{constraints}\\ r(x(t_{f}))=0 & & \text{terminal}\;\text{constraints} \end{array}$$

where ***u*** are the *inputs* or *controls*, *t_f_* the time over which the controls are exercised, ***x*** the states, *l* the *running cost*, e,f,h,r are known functions based on the specific problem and $x_{0}$ is the initial condition at time *t* = 0. The state $x\in R^{n_{x}}$ corresponds to the voxel concentrations in a grid in the region of interest. Thus the concentration distribution at the initial time is $x_{0}.$ This region is surrounded by *n_u_* electromagnets with their associated controllable currents $u(t)\in R^{n_{u}}.$ The dynamical model is included in *f*. Herein the behavior of the particles developed in previous section is included $\dot{x}=f(x,u)=A(u)x,$ see (5). Path constraints are set to limit the currents to a maximum value imposed by the coil conductor properties: h(x,u)=− abs$(u)+u_{\max}\geq 0.$ Here abs(·) is the element-wise absolute value. Various expressions for the running cost l(x,u) are applicable, as shown with the following examples. To target or avoid certain locations containing many or few particles, the particle concentration in the corresponding voxels should be as large or as small as possible, which translates to a maximization or a minimization of these voxel concentrations. This yields the term $x^{T}Qx$ where $Q\in R^{n_{x}\times n_{x}}$ “selects” the voxels of interest by taking the diagonal elements either a positive or a negative value with magnitude depending on the weight or importance of the chosen voxel. Additionally, the power dissipated by the electromagnets can be minimized by including $u^{T}Ru$ in the cost function, $R\in R^{n_{u}\times n_{u}}$ a diagonal matrix. Lastly, if one aims to minimize the time in which a certain condition is met, the final time *t_f_* is an optimization variable. The total cost function *J* is then, summarized,

(7) $$J=\int_{0}^{t_{f}}l(x(t),u(t))dt+e(x(t_{f}))=\int_{0}^{t_{f}}\left(x^{T}Qx+u^{T}Ru\right)dt+\gamma t_{f}$$

where *γ* is a weight to be chosen in agreement with the relative importance of the final time. As discussed in Komaee & Shapiro (2011), one could also add the spreading of a distributed ferrofluid as a cost function. The lower the spreading, the more particles are at the target location.

Approaches to solve the optimal control problem are dynamic programming, indirect methods and direct methods (Diehl et al., 2006). In dynamic programming, the computationally expensive operation of solving the Hamilton-Jacobi-Bellmann (HJB) equation is required and as such it is limited to small state dimensions, e.g. when a smaller sample or a coarser resolution need to be used. Indirect methods imply the construction of adjoint equations and are generally difficult when it comes to dealing with constraints. Because direct methods are more readily set up and solved than indirect methods, a direct method is used in this work, more specifically the method of *direct multiple shooting* (DMS). DMS is useful compared to other direct methods, as it allows to work with state-of-the-art adaptable ODE solvers that are at our disposal. The output of the DMS algorithm are the electromagnet currents as a function of time, discretized in piecewise constant levels. For example, when the number of time steps or levels *n_t_* is 4 with a total coil excitation time of 10000 s, then each 2500 s a different constant current value is imposed. The more time steps considered, the longer the calculation and the more accurate the result. A detailed explanation of the DMS algorithm is beyond our scope, but we refer to (Bock & Plitt, 1984; Diehl et al., 2006; Nocedal & Wright, 2006). In-house MATLAB code is used in our simulations.

Since the number of states (here: the number of voxels) *n_x_* and time steps *n_t_* greatly affect the computational burden when solving the DMS problem, the simulation time to find optimal trajectory inputs quickly becomes unacceptable when they reach a certain level. This implies that too few voxels could be considered. For example, DMS applied to a 20 × 20 voxel grid and 5 time steps did not finish computing after more than 5 h. To deal with this problem, the method of model order reduction is introduced.

### 2.4. Model order reduction

The aim of model order reduction is to reduce the computational burden of the numerical simulation of a system. By constructing a so-called *reduced-order model* with a lower dimension than the original model’s (*full-order model*) dimension, the evaluation time can be drastically decreased (Lassila et al., 2014). In order to transform a state-space model $\dot{x}=f(x,u),x\in R^{n_{x}},$ to a reduced model $\dot{\tilde{x}}=\tilde{f}(\tilde{x},u),\tilde{x}\in R^{\ell },\ell \ll n_{x},$ one may use the method of *proper orthogonal decomposition* (POD) (Volkwein, 2013). In this method, the so-called *snapshot matrix X* of the original dynamical system is introduced. This is a matrix containing the solution of $\dot{x}=f(x,u)$ at different time instances in a certain time interval [0,*t_f_*]

$$X=[x(t_{1}),\ldots ,x(t_{n_{s}})]\in R^{n_{x}\times n_{s}}$$

*n_s_* is the number of snapshots and rank $\left(X)=d\leq \min\{n_{x},n_{s}\right\}.$ The aim is to find a reduced number of basis vectors by which the snapshots can be expressed (Schilders et al., 2008). This is accomplished by means of the singular value decomposition of *X*

$$X=U\Sigma V^{T}.$$

The *i*-th column of *U* is written as $g_{i}$ so $U=[g_{1},\ldots ,g_{n_{x}}].$ It can be proven that for every ℓ≤d the approximation of the columns of *X* by the first ℓ singular eigenvectors ${\left\{g_{i}\right\}}_{i=1}^{\ell }$ is optimal in the mean among all rank ℓ approximations to the columns of *X*. These vectors ${\left\{g_{i}\right\}}_{i=1}^{\ell }$ are called POD basis of rank ℓ. Hence $U_{\ell }=\text{span}\left\{g_{1},\ldots ,g_{\ell }\right\}$ is an optimal projection space of dimension ℓ that captures most of the dynamical behavior of the original system. The reduced-order model is created by approximating ***x*** in $U_{\ell }$ as

$$x(t)\approx U_{\ell }\tilde{x}(t),\tilde{x}(t)\in R^{\ell }$$

with $U_{\ell }=[g_{1},\ldots ,g_{\ell }].$ The reduced system is then $\dot{\tilde{x}}=U_{\ell }^{T}f(U_{\ell }\tilde{x},u),$ or, if f(x,u)=A(u)x,

$$\frac{d\tilde{x}(t)}{dt}=U_{\ell }^{T}A(u(t))U_{\ell }\tilde{x}(t)=\tilde{A}(u(t))\tilde{x}(t)$$

with arguments. Two steps are identified for obtaining the reduced-order system:

1. Select appropriate snapshots in the time grid to obtain *X*. A rigorous optimal selection of snapshots is not straightforward. The reader is referred to Kunisch & Volkwein (2010) for more information. The snapshot matrix is acquired from a numerical solver which is used for solving Equation (5).
2. Choose ℓ. It is clear that, if ℓ is chosen small, the dimension of the problem is decreased significantly, but the ability to capture the full dynamics is also diminished. A measure for the approximation error is *ε*
  (8) $$\varepsilon (\ell )=\frac{\sum_{i=1}^{\ell }\sigma_{i}^{2}}{\sum_{i=1}^{d}\sigma_{i}^{2}}$$

where $\sigma_{i}=\Sigma_{ii}$ are the singular values of *X*. One determines a suitable ℓ heuristically based on ε(ℓ), which should be close to 1. For small ℓ, less POD modes are considered and the reduced system is less accurate. The more POD modes that are considered, the closer the solution is to the original solution. There is no theoretical lower bound for *ε* (Volkwein, 2013).

The goal is to alleviate the computational burden of the dynamic optimization by reducing the number of target variables (Alla & Falcone, 2013; Schmidt, 2014). The optimal control problem (Equation (6)) is rewritten in terms of the reduced-order variable $\tilde{x}=U_{\ell }^{T}x.$ The cost functional reads

(9) $$\tilde{J}(\tilde{x},u)=\int_{0}^{t_{f}}\tilde{l}(\tilde{x}(t),u(t))\;dt+\tilde{e}(\tilde{x}(t_{f}))$$

with $\tilde{l}(\tilde{x},u)={\tilde{x}}^{T}\tilde{Q}\tilde{x}+u^{T}Ru=x^{T}Qx+u^{T}Ru$ with $\tilde{Q}=U_{\ell }^{T}QU_{\ell }$ and equivalently the constraint functions are transformed with respect to the reduced-order states. It is important to note that the result of this optimization will be different with respect to the result of the optimization for another POD basis, thus another $U_{\ell }.$ Therefore, it is useful to run *multiple* optimization sequences with a judicious choice of $U_{\ell }.$ Afterwards, the optimal candidate for the full-order system may be chosen. The initial current guess to the dynamic optimization can be the result of a fast local optimization routine for focusing ferrofluids developed in prior research (Nacev et al., 2012). With this current sequence applied to the setup, a snapshot matrix *X* of the full-order states can be computed, which then allows to find a suitable ℓ and $U_{\ell }.$ Repeating this sequence multiple times may further enhance the end result of the optimization, as outlined in the following algorithm similar to what can be found in Baumann (2013). The dynamic optimization is run for a limited number of iterations to find reduced-system controls. The controls corresponding to the optimal state trajectory are applied to the original full-order system to obtain snapshots. These new snapshots are then used to find a new POD basis. Starting from the previous optimal inputs and resulting discretized initial states, a new gradient-based nonlinear programming sequence is executed to find an optimal control. Schematically:

### 2.5. Algorithm for 2-D dynamic optimization in pseudo-code

1. Set initial guess zero-order hold current sequence $u_{g},$ *i* = 0, and number of dynamic optimization sequences $n_{\text{opt}}$
2. Solve the discretized advection-diffusion PDE $\dot{c}=A(u_{g})c$ to obtain $c^{\left(0\right)}$
3. **while** $i\leq n_{\text{opt}}$ or ‘current target voxel concentration(s) < desired target voxel concentration(s)’

Compute the POD modes from the snapshots ${\text{c}}^{\left(i\right)}$ to obtain $U_{\ell }^{\left(i\right)}$

Run the dynamic optimization (DMS) of the reduced system with cost function (9) and $\tilde{c}=U_{\ell }^{(i)T}c$

Exit the optimization sequence and obtain the controls $u^{\left(i\right)}$

Solve the full-order advection-diffusion PDE $\dot{c}=A(u^{\left(i\right)})c$ and assign the resulting snapshots to $c^{\left(i+1\right)}$

i=i+1

**end**

## 3. Results and discussion

To validate the presented algorithm, it is applied to the setup discussed in Section 2.1. We consider two scenarios: one in which a concentrated particle distribution is guided toward voxels of interest, and a second in which a concentrated ferrofluid, in a uniform blood flow, is either kept at its initial position or moved.

The cost function for these scenarios is formulated, in agreement with (9), as

$$\tilde{J}(\tilde{x},u)=\int_{0}^{t_{f}}{\tilde{x}}^{T}U_{\ell }^{T}QU_{\ell }\tilde{x}\;dt$$

where the diagonal element of *Q* corresponding with the target voxel is −1 and zero elsewhere, *R* = 0 and *γ* = 0 in (7). Only the voxel concentration and not the final time nor the dissipated energy are optimized for in this analysis, because the weights of final time and energy depend heavily on the specific experimental circumstances. In all cases, the maximum coil current is 13 A. If required, upper and lower bounds can be set for concentrations in voxel(s) of choice, for example to avoid high particle concentrations at the domain boundaries. This corresponds to the path constraint function *h* in (6), in the reduced system $\tilde{h}(\tilde{x},u)=h(U_{l}\tilde{x},u).$ The total excitation time *t_f_* is here chosen to be 10000 s. This rather large value is only an indication and a direct consequence of the size of the setup, the used particles, and phenomena such as particle agglomeration. The total excitation time can be reduced under different circumstances. An experimental study of the wide range of magnetic particle speeds under magnetic gradients was conducted in Benhal et al. (2019). Targeting times of 1 h of applying magnetic sources have been reported in *in-vivo* studies (Muthana et al., 2015; Al-Jamal et al., 2016).

The initial distribution of nanoparticles is comprised in a single region, e.g. by activating a single magnet and capturing the particles as close as possible to the magnet and a wall or membrane that is impenetrable for the particles. This distribution is known to us (by imaging) and is the initial condition $x_{0}$ for the optimal control algorithm. Next, the number of time steps *n_t_* and the number of reduced-order states ℓ have to be determined. ℓ is obtained from setting e.g. ε=0.98 in (8). The larger ℓ, the more likely the capturing of the full system dynamics is accurate. A representation of the effect of *n_t_* and ℓ on the computational time on a laptop pc (Intel Core i7, 12 GB RAM) of one optimization sequence is given in Table 1. In all the following simulations, *n_t_* = 4 and the sample grid size is 31 by 31 voxels, which amounts to 961 full-order states (the voxel concentrations). From (8) we obtained ℓ=6.

**Table 1.** computational time for different *n_t_* and ℓ for one optimization sequence.

*n_t_*	ℓ	comp. time [min.]
4	6	5
4	60	37
4	961 (full)	≫
20	6	153
20	60	≫

The increase in computational time when more time steps (*n_t_*) are considered is, apart from there being more optimization variables, also a consequence of the increased number of times that the magnetic fields and forces need to be calculated.

The DMS algorithm requires an initial guess of input currents with respect to time. This initial guess is set based on setup parameters and *a priori* knowledge of the setup. The direction of the motion allows to determine which coils are preferred to be activated. Once this initial guess is determined, the DMS algorithm can be run.

### 3.1. Steering concentrated particle ensembles toward a single spot in a stationary fluid

**Figure 2.**
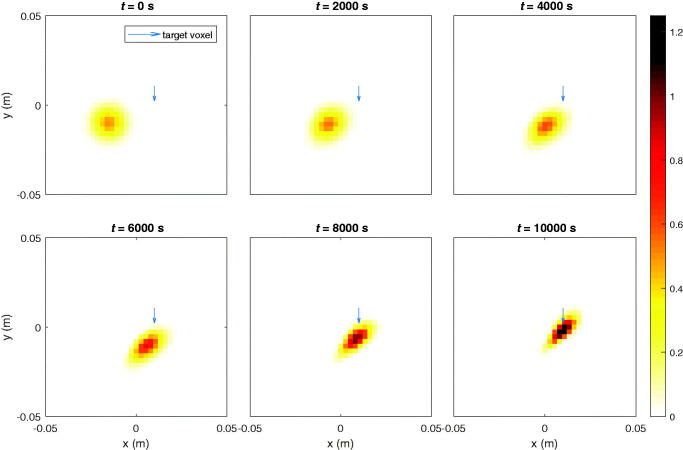
Ferrofluid guided toward target voxel (blue arrow) at [0 cm; 1 cm] by maximizing its particle concentration.

**Figure 3.**
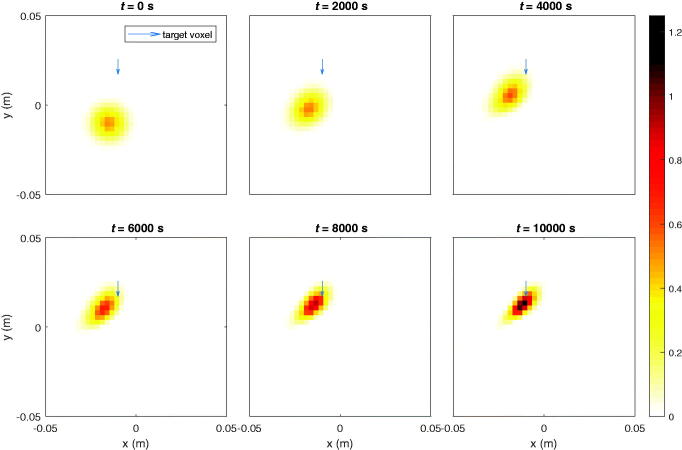
Ferrofluid guided toward other target voxel (blue arrow) at [-1 cm; 1.5 cm] by maximizing its particle concentration.

**Figure 4.**
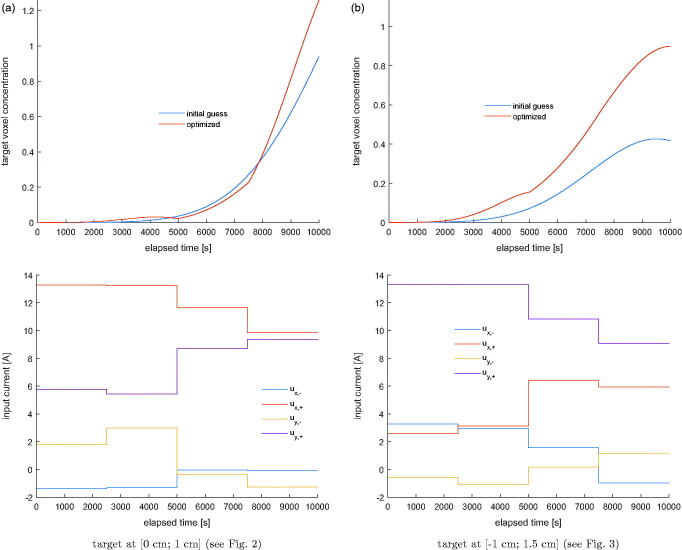
The voxel particle concentration for (a) the first and (b) the second target. The optimization may be terminated as soon as a feasible concentration level is reached within the predefined time interval.

In this scenario an ensemble of particles is steered toward a predefined voxel. At 4 time instances, a snapshot of the sample with the concentration distribution of particles in the stationary liquid with the viscosity of blood is taken, which are given in Figure 2. The single target voxel is indicated by the blue arrow. Figure 3 shows another example in which a different voxel is set as the target. The particle concentration in the target voxel with respect to time and the accompanying currents for both cases are shown in Figure 4. The particle concentration numbers end up being significantly improved compared to the initial guess.

In reality, the current signal takes a finite time to change current levels, but this is negligible compared to the particle velocities. The currents are constrained to a maximum absolute value imposed by the coil specifications and available cooling capacity.

These results show clearly that no optimal predefined trajectory of the particles is required to focus particles around a voxel, while taking into account the evolution of the particles in the whole time interval. This is useful when liquid flow rates different from zero are present, because in this case it is not known beforehand which optimal trajectory the particles should follow to be brought toward the target.

### 3.2. Moving or holding concentrated particle ensembles in a moving fluid

In the following results, it is shown that the algorithm can deal with fluid velocity profiles. When flow velocity comes into play, the feasibility to focus particles at certain points may decrease significantly, depending on the attainable magnitude of the magnetic forces. Too strong fluid drag against too low magnetic forces will make the dynamical system lose controllability. Moreover, the presence of fluid velocities makes the targeting accuracy more susceptible to errors in the controls.

**Figure 5.**
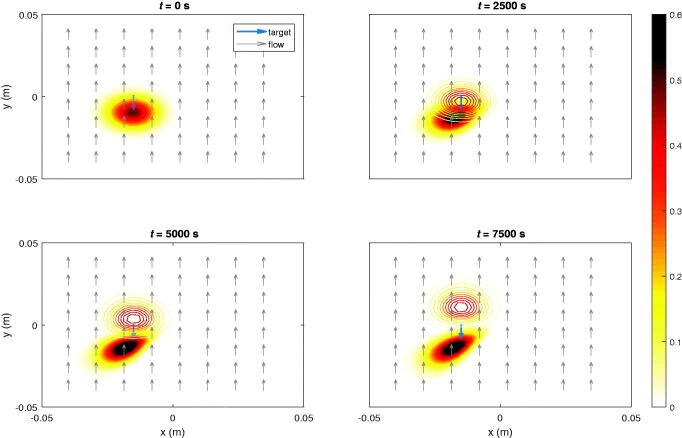
Ferrofluid controlled at initial location (blue arrow). The contours show the hypothetical evolution of the ferrofluid if no magnetic fields would have been applied. The flow is uniform throughout the sample and is represented by gray arrows.

**Figure 6.**
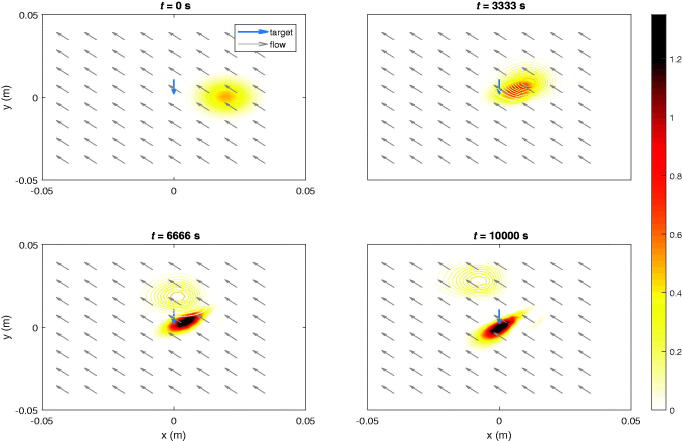
Ferrofluid guided to target (blue arrow) in uniform diagonal flow velocity. The contours show the hypothetical evolution of the ferrofluid if no magnetic fields would have been applied. The uniform diagonal flow velocity is indicated by gray arrows.

A uniform vertical fluid velocity, *v_fy_* = 3 µm/s, is present in Figure 5. The fluid drag force significantly impacts the movement of the particles. To maintain the ferrofluid at the initial location, against the fluid stream, the target voxel is the center of the initial distribution. If no fields were applied, the particle would move along with the flow. This procedure can be used to keep particles focused e.g. at a diseased location for a necessary period of time against body flow drag, which would not be possible with prior methods that require a predefined trajectory in a stationary liquid. This hypothetical movement is depicted by contours. In Figure 6, the particles are manipulated toward the center in a uniform diagonal flow velocity, i.e. $v_{fx}=-3$ µm/s, *v_fy_* = 3 µm/s. These values are chosen with respect to what is feasible with our parameters (the particles traveled approximately 3 cm in 10000 s in the previous simulations).

**Figure 7.**
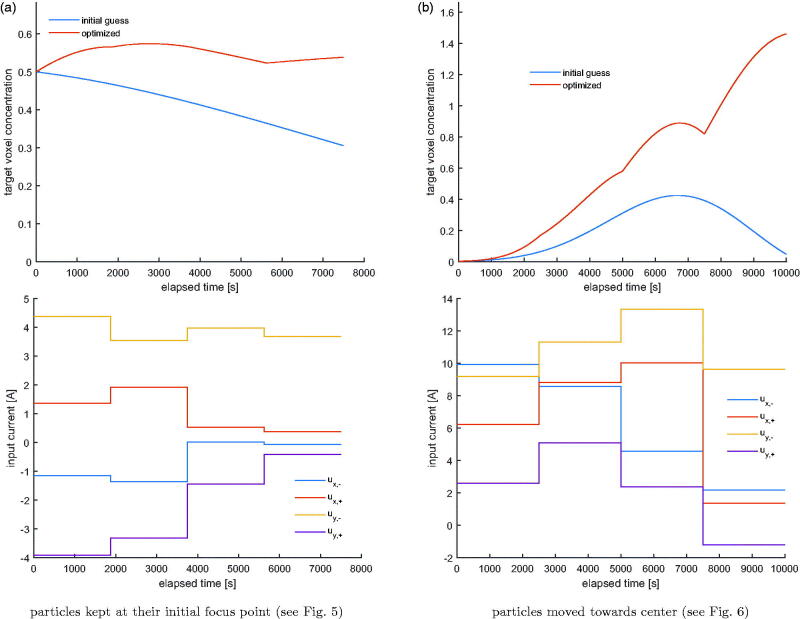
The voxel particle concentration for (a) the first and (b) the second target. The optimization may be terminated as soon as a feasible concentration level is reached within the predefined time interval.

Lastly in Figure 7, the concentration of MNPs in the target voxel indicated in Figures 5, 6 and current signals are plotted with respect to time in the same manner as in previous section.

### 3.3. Remarks

The results above demonstrate that particle collections can be successfully guided toward a voxel of choice by dynamically updating the electromagnet currents and thus the magnetic forces using the proposed methodology, both with and without fluid flow velocity. The duration of the control calculation was about 23 min. This computation time can be reduced when a smaller number of voxels is considered, or when the number of time steps and ℓ is smaller, or by simply utilizing better hardware. Also, it is possible to target a voxel between the initial distribution and the target over a shorter time period, and then repeat this until the target voxel is reached, reducing the overall computation time.

As already mentioned, the real-time duration of the ferrofluid movement (10000 s ≈ 3 h) is governed by behaviors and setup parameters such as particle size, magnetic fields, fluid viscosity and diffusion rate. Upon choosing a fixed finite time interval and target voxel(s), it is very well possible that the combination of e.g. particle size and available magnetic fields does not allow the particles to move as far as the predefined target. In that case, any optimization over a fixed time will not converge toward a satisfactory distribution as it is physically unachievable.

The controls and state trajectory are calculated in a *feedforward* way, meaning that they are purely based on modeled behaviors without feedback from real-time measurements during the motion. This makes the strategy susceptible to modeling errors. Future research may focus on estimates of model errors and the inclusion of particle location information from a camera or other imaging technique to better correct for them.

The targeting strategies reviewed by Nacev et al. (Shapiro, 2009; Nacev et al., 2012) perform an optimization of the instantaneous distribution on single time instances without taking into account future behavior or maximizing target concentrations, whereas our optimization is over a full time period and targets a voxel of interest. Komaee discussed the use of optimal control for minimizing the particle spread while moving along a piecewise differentiable trajectory that is defined beforehand (Komaee, 2017). Our algorithm does not require the prior definition of such a trajectory, because the optimization inherently searches the best curve along which the target voxel concentration is increased. This is an advantage, as the user most of the time has no knowledge of the optimal path of the particles, especially against a moving fluid.

As is clear from the results, our control algorithm can steer particles in biological environments while preventing them from being scattered or washed away by fluidic forces and diffusion mechanisms. Moreover, the voxel-based modeling approach can be integrated easily with imaging modalities. Target voxels can be selected to maximize their concentration of particles, which is important when certain locations require high levels of (nano)particles for improved therapy. We furthermore believe that the introduction of model order reduction techniques was highly necessary to achieve the minimum levels of accuracy in such applications. This can be exploited further in future control algorithms.

Lastly, guiding/targeting performance and energy cost can be enhanced by alterations to the (physical) setup. For example, adding more coils around the sample increases the accuracy of the magnetic fields. This can lead to improved focusing and reduced energy consumption, or opens the possibility of 3-D targeting, at the cost of a more complex setup.

## 4. Conclusion

One of the challenges of effective magnetic drug targeting is the guiding and deep tissue focusing of magnetic nanoparticle collections in the human body with external magnetic fields from electromagnets. No such experiments have been conducted on human patients, but *in-vitro* tests and simulations have shown the potential of dynamic control to achieve this goal (Nacev et al., 2012; Shapiro et al., 2015). That targeting challenge was addressed in this paper by developing a dynamical model and a model-based control algorithm.

The dynamical model is developed by combining magnetic and fluid forces acting on the particles in fluid environments and advection-diffusion mechanisms. By writing the dynamical equations with respect to the voxel concentrations, an evaluation of the concentration in every voxel with respect to time becomes possible. This is very useful if put next to imaging modalities which often aim to reconstruct the voxel concentrations from measurements, applicable in theranostic platforms. The resulting ODE is immediately applicable to optimal control algorithms.

In optimal control, a certain control or input is searched for a dynamical system over a period of time that minimizes an objective function. We have linked the different parts of a general optimal control problem with the ones of the magnetic targeting problem. The feedforward controls are the currents in electromagnets – they directly determine the magnetic forces – and the objective function is the concentration in the targeted voxel. Constraints on the currents and concentrations can be added. To solve this problem numerically, the method of direct multiple shooting was the best option. Such a dynamic optimization may take unacceptable computation times because of the large number of variables. This is addressed by reducing the number of states using techniques drawn from the field of model order reduction. A reduced model is used in the optimizer, and once the controls are calculated, they can be applied to the full-order model.

The control strategy was tested for a 2-D MDT setup consisting of 4 coils, in which two scenarios were considered: stationary and non-stationary fluids. The simulation results showed that particle ensembles in the fluid medium were effectively guided toward and focused around a predefined voxel of interest by varying magnetic fields, which is one of the ultimate goals of MDT. Moreover, the objective function can easily be changed to include more voxels or time and energy requirements weighted according to the needs of the user, who benefits from this increased flexibility.

Experiments in a lab setup need to be done to verify and improve the algorithm with real-time measurements. Future research may elaborate on this with a more sophisticated model taking into account *in-vivo* vasculature systems and intervening blood flow forces in the body, more versatile targeting setups (extension to 3-D), communication between targeting and imaging sequences, etc. With the presented methodology we have taken steps to progress MDT performance for future (pre)clinical research of particle-based targeting applications.
